# Supplementary material for: An evaluation of the self-assembly enhancing properties of cell-derived hexameric amyloid-β
Source: Sci Rep. 2021 Jun 2;11:11570. doi: 10.1038/s41598-021-90680-y (PMC8172837; doi:10.1038/s41598-021-90680-y)
Supplement: Supplementary file 1 — Supplementary Information. [file 41598_2021_90680_MOESM1_ESM.pdf]

Supplemental Data

An evaluation of the self-assembly enhancing properties of cell-derived hexameric amyloid-β

Devkee M. Vadukul[1,2†], Céline Vrancx[1], Pierre Burguet[3], Sabrina Contino[1], Nuria Suelves[1], Louise C Serpell[4], Loïc Quinton[3] and Pascal Kienlen-Campard[1*]

[1]Alzheimer Research Group, Molecular and Cellular division (CEMO), Institute of Neuroscience, Université catholique de Louvain, Brussels, Belgium.

[2]Molecular Sciences Research Hub (MSRH), Department of Chemistry, Imperial College London, London, UK

[3]Mass Spectrometry Laboratory, MolSys Research Unit, University of Liège, Liège, Belgium

[4]Sussex Neuroscience, School of Life Sciences, University of Sussex, Falmer, Brighton, East Sussex BN1 9QG, UK

[†]Current affiliation

***Corresponding author:**

Pascal Kienlen-Campard (pascal.kienlen-campard@uclouvain.be)

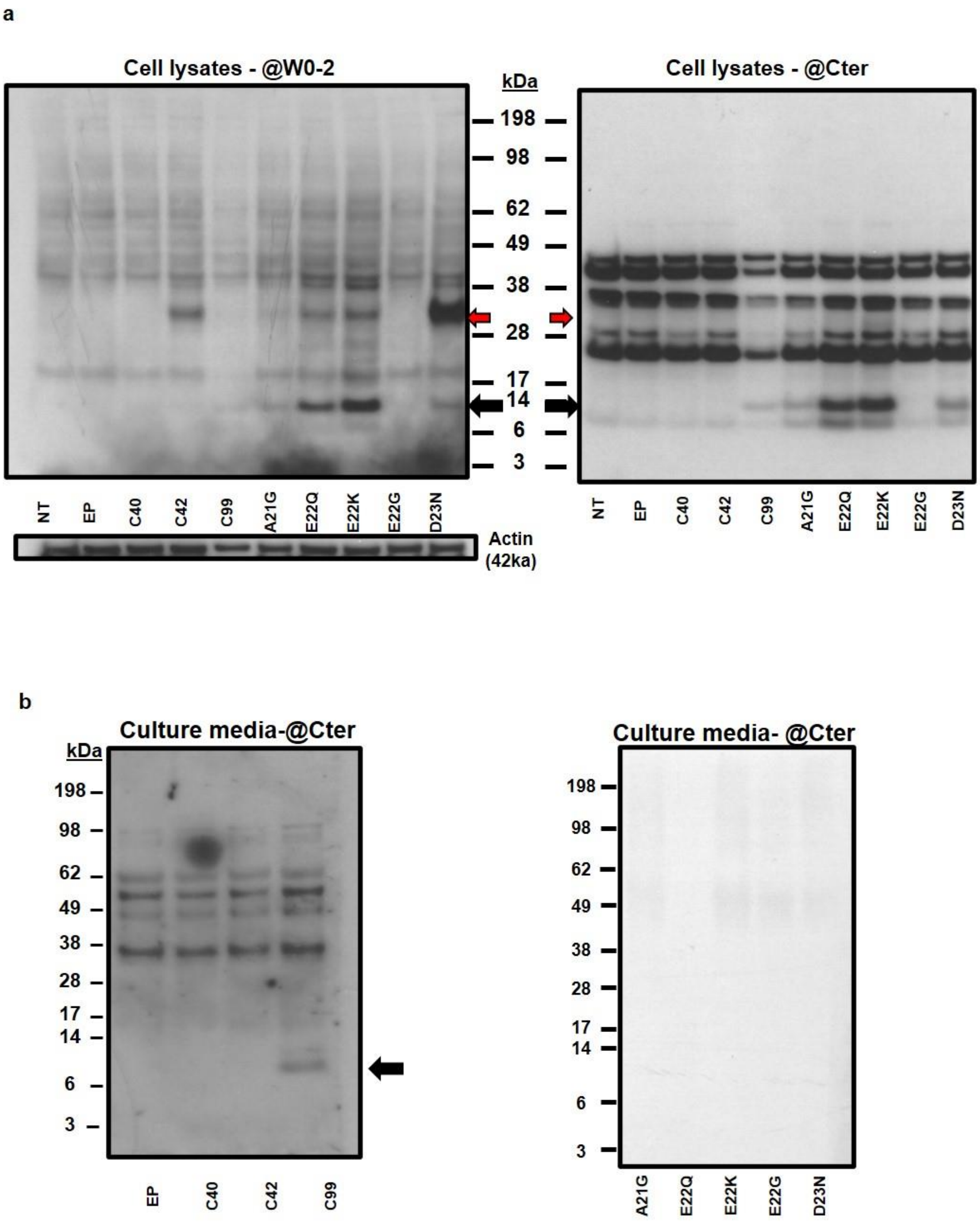

Supplemental Figure S1. APP C-terminal detection with Cter antibody confirms assemblies to be Aβ. Hexameric assemblies are not detected by the Cter antibody in the cell lysates (a) or culture media (b) of CHO cells transfected with EP, C40, C42 or C99 as well as FAD mutants. The C-terminal fragment is seen in the cell lysates of CHO cells transfected with C99 sequences (black arrows). A side-by-side comparison of anti-W0-2 and anti-Cter detected Western blot of cell lysates (a) shows that although a similar band to the hexamer (red arrow) is detected by the anti-Cter antibody, this is lower than the band detected by W0-2. Actin (42kDa) loading control is provided for the cell lysate conditions.

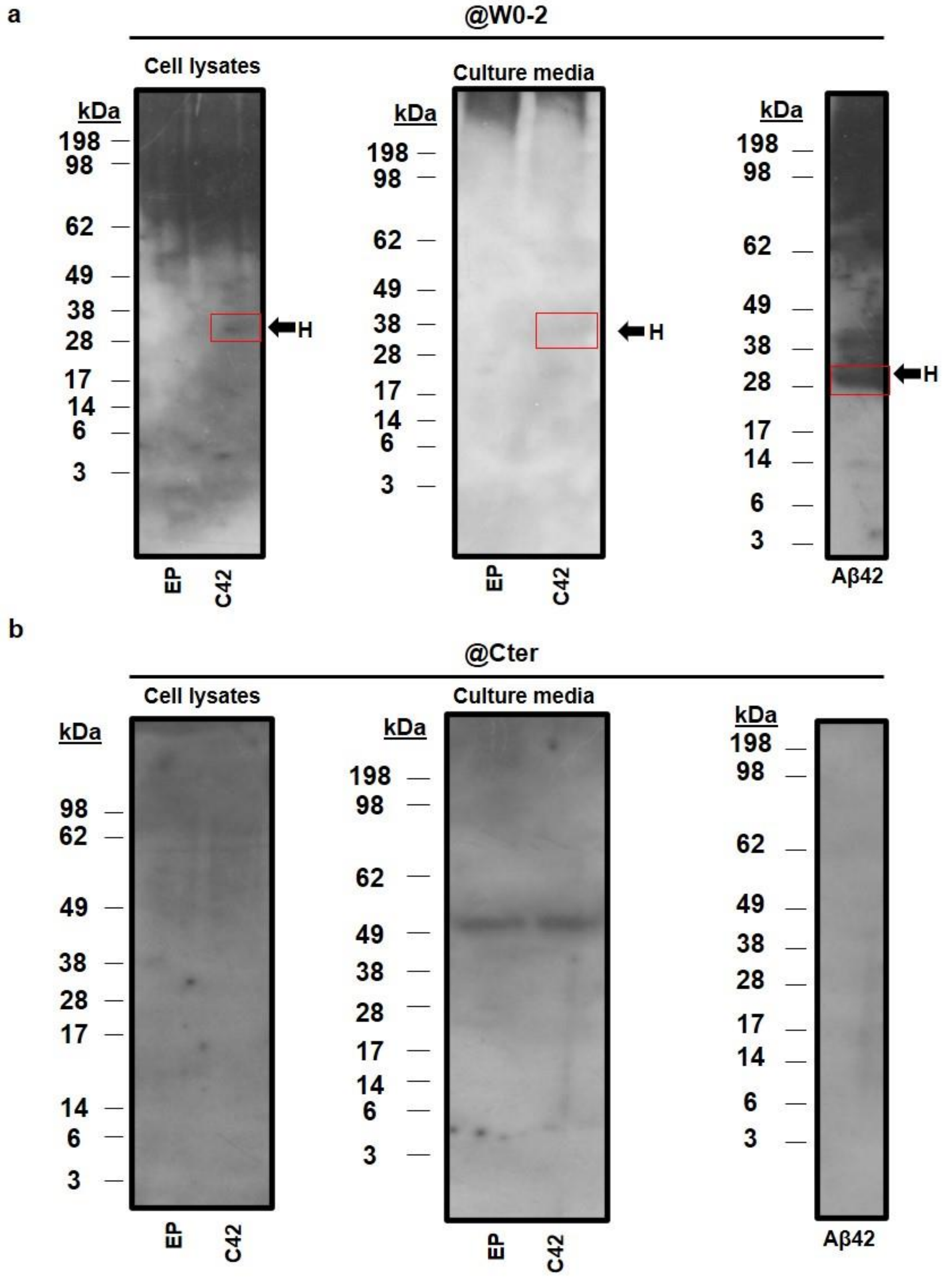

Supplemental Figure S2. Identified hexamers are not an artifact of SDS. (a) Hexameric assemblies were detectable by the W0-2 antibody in CHO cell lysates and media samples (left and middle panels) that were harvested and analysed in SDS-free conditions. Furthermore, synthetic Aβ42 preparations (right panel) also showed the presence of this hexameric band in SDS-free conditions. (b) The anti-Cter antibody did not detect these hexameric bands from the same samples.

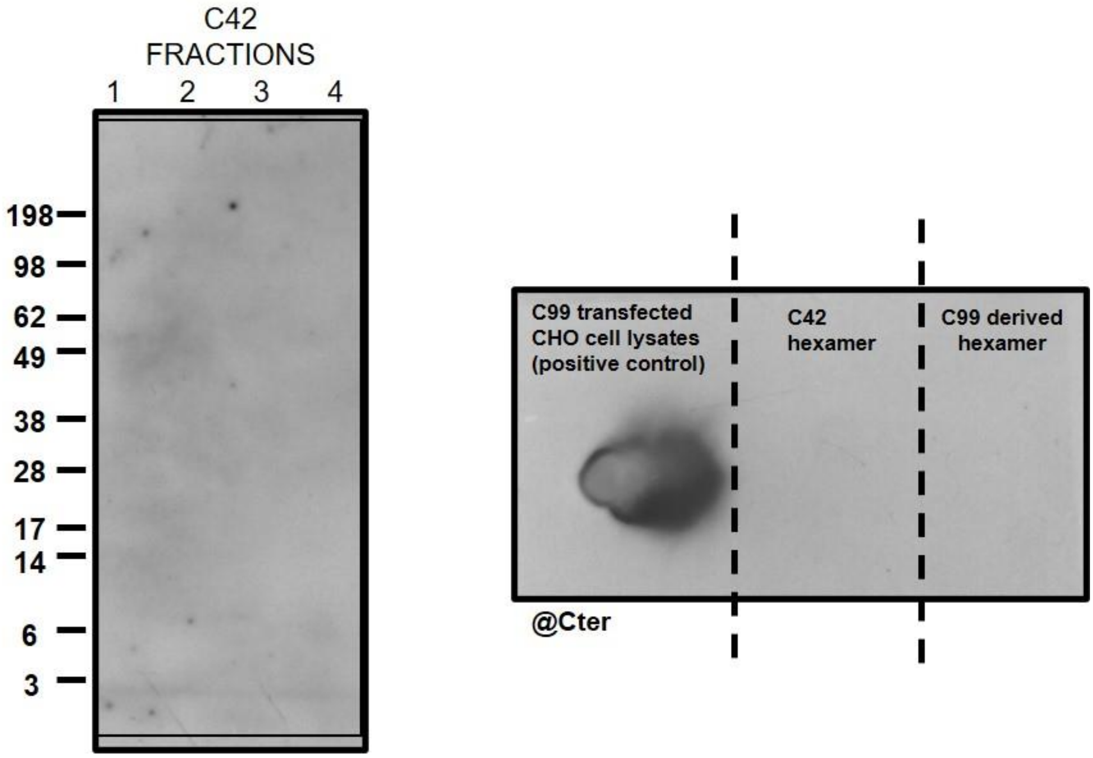

Supplemental Figure S3. Isolation of C42 derived hexamers: Fractions 1-4 and anti-Cter dot blot for isolated hexamers. (Left panel) Gel edges indicated by thin black outline. No Aβ assemblies were isolated in fraction 1-4 collected after separation by the GELFrEE 8100 system and assessed by Western blotting detected with the anti-Cter antibody. (Right panel) Dot blot detected with the anti-Cter antibody does not detect either of the isolated hexamers. C99 transfected CHO cell lysates were used here as a positive control. Dashed lines represent distribution of samples on membrane.

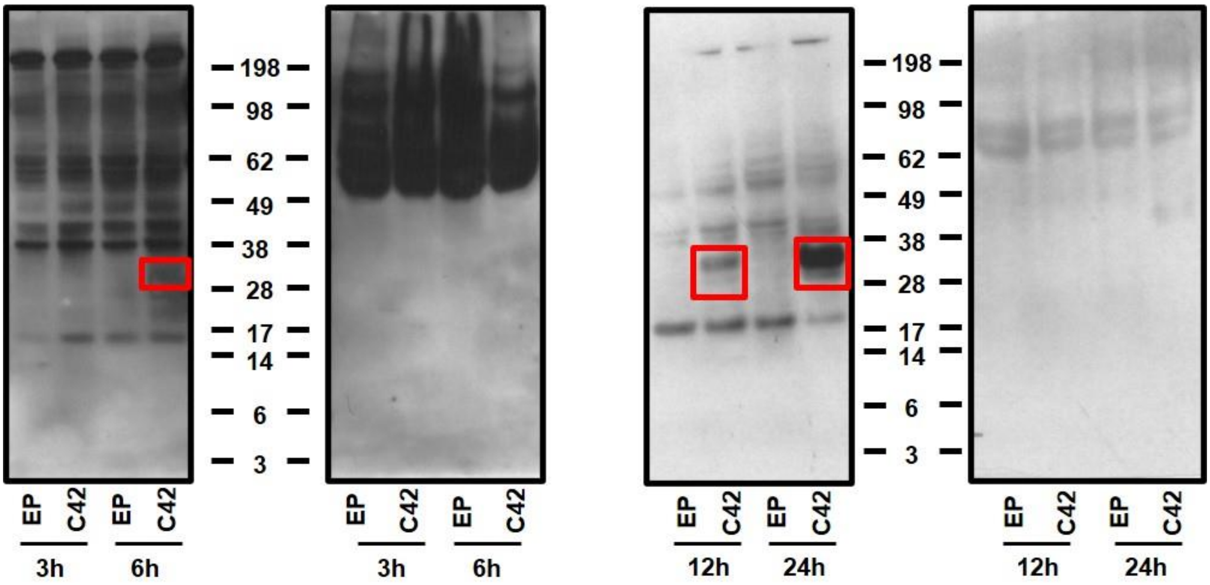

Supplemental Figure S4. Lower molecular weight Aβ assemblies were not detected in C42 transfected CHO cells. CHO cells were transfected with C42 for 3,6,12 and 24hours. Formation of hexameric assemblies were seen in cell lysates by 6 hours with no lower weight assemblies seen at the earlier time point of 3 hours. Aβ assemblies were not detected in the media of these cells even after 24 hours. Western blot detection with the W02 antibody.

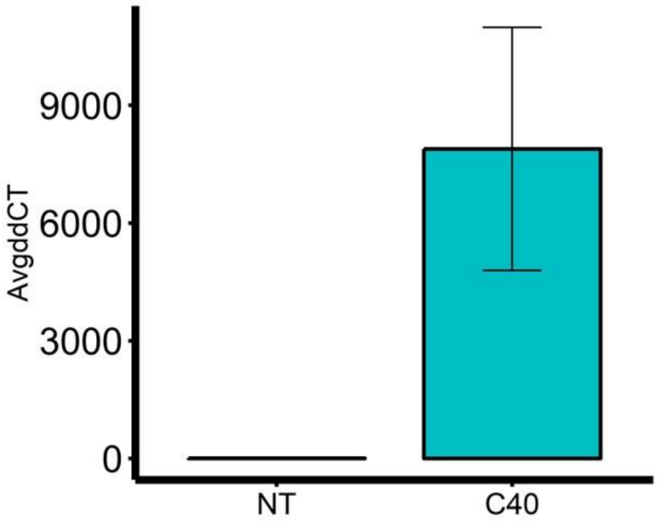

Supplemental Figure S5. C40 transfection efficiency. qPCR confirms that the lack of hexameric assemblies is not due to low transfection efficiency (N=5)

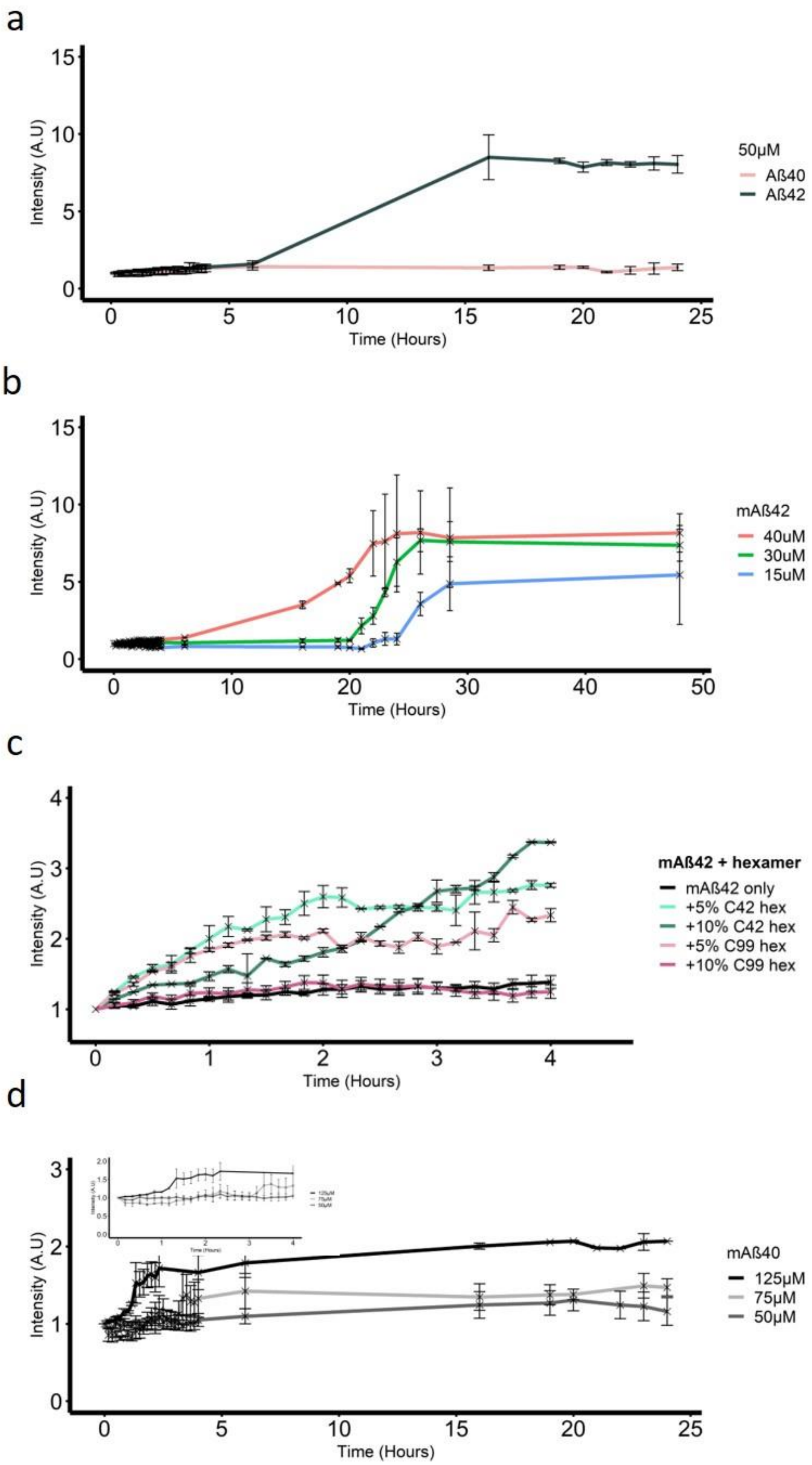

Supplemental Figure S6. ThT fluorescence of Aβ40 and Aβ42 over time. (a) 50μM of each peptide was incubated with 20μM ThT and fluorescence intensity was measured over 24hours. Aβ40 does not display an increase in fluorescence however Aβ42 displays a lag phase (T0-4h), elongation phase (4h-16h) and a plateau. (b) ThT aggregation assay of a range of Aβ42 concentrations show a concentration dependent aggregation. (c) ThT aggregation assay to show additional data points in Aβ42 hexamer seeded conditions from T0-4h (d) ThT aggregation assay to show Aβ40 does aggregate in 24h at higher concentrations. Inset: zoom on ThT fluorescence for clarity between T0-4h.

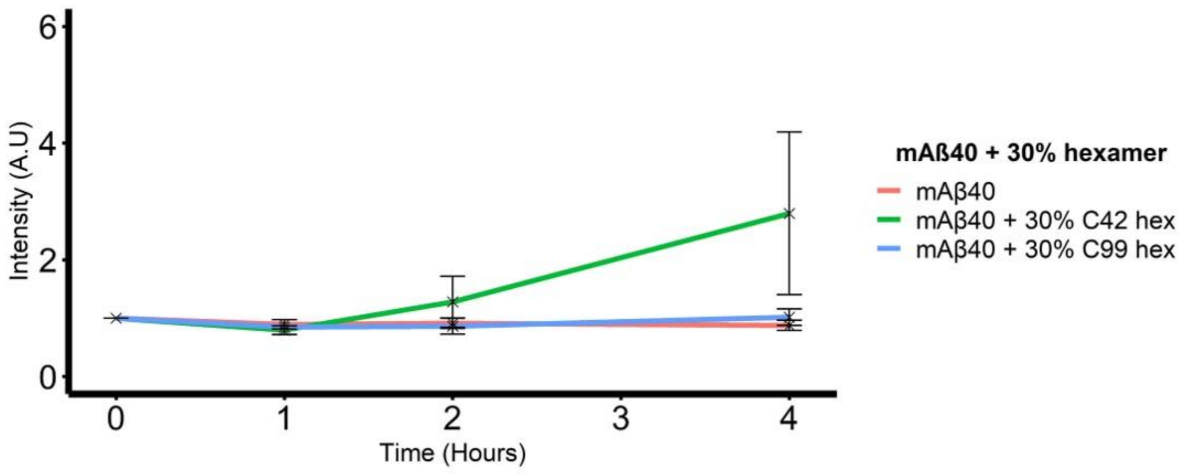

Supplemental Figure S7. Monomeric Aβ40 does show accelerated aggregation after 30% hexamer addition. ThT aggregation assay to show that with 30% addition of hexamer, an enhanced self-assembly of Aβ40 does occur over 4h.

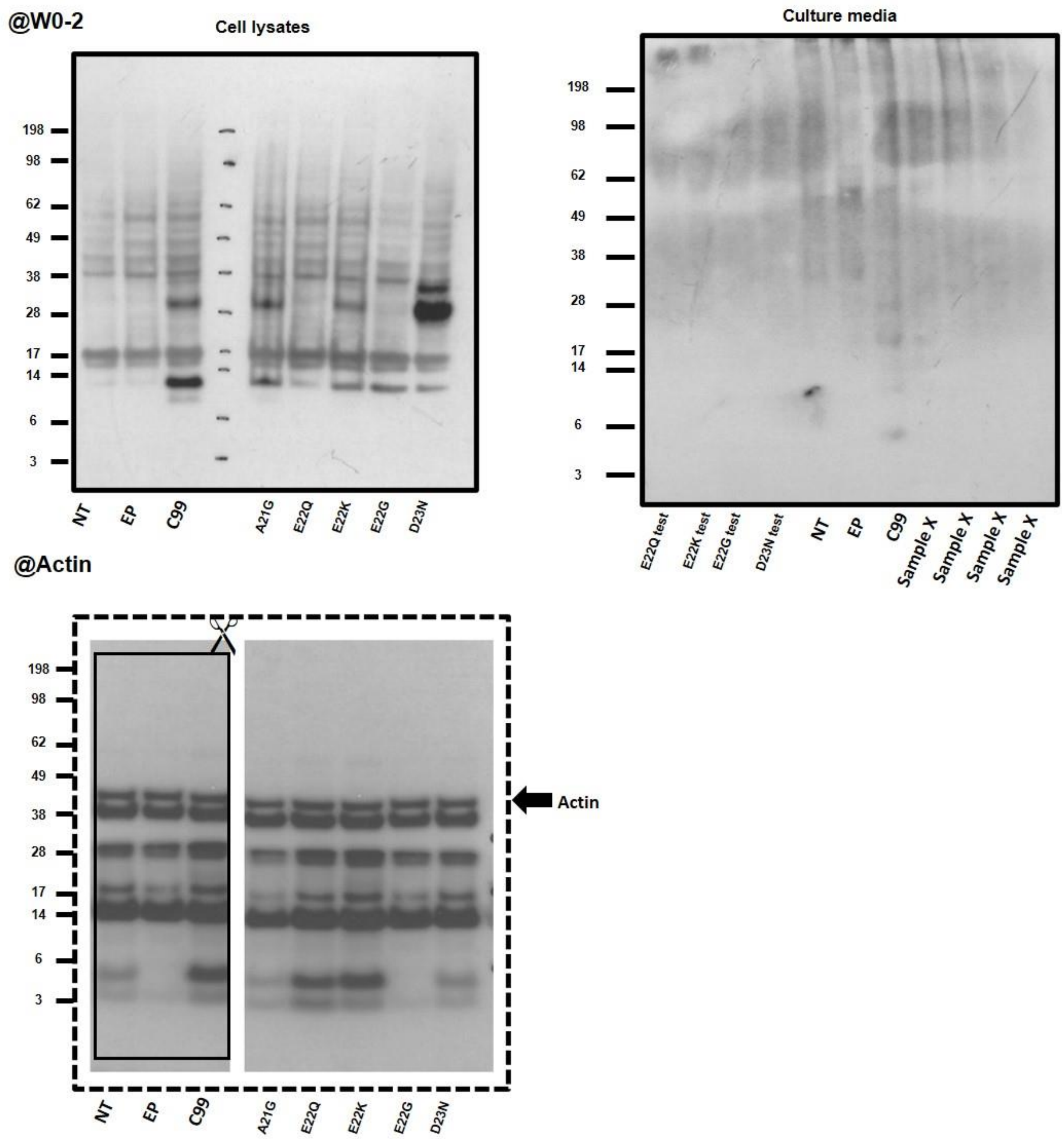

Supplemental Figure S8. Full length original gels of the cropped images presented in Figure 1a. For the Actin detection, a solid black border indicates the edges of the membrane for samples presented in Figure 1a only; dashed lines indicate that the detection film was cut to separate samples before scanning, however these have been combined here to represent the full length original membrane. Sample X are data not presented in the manuscript.

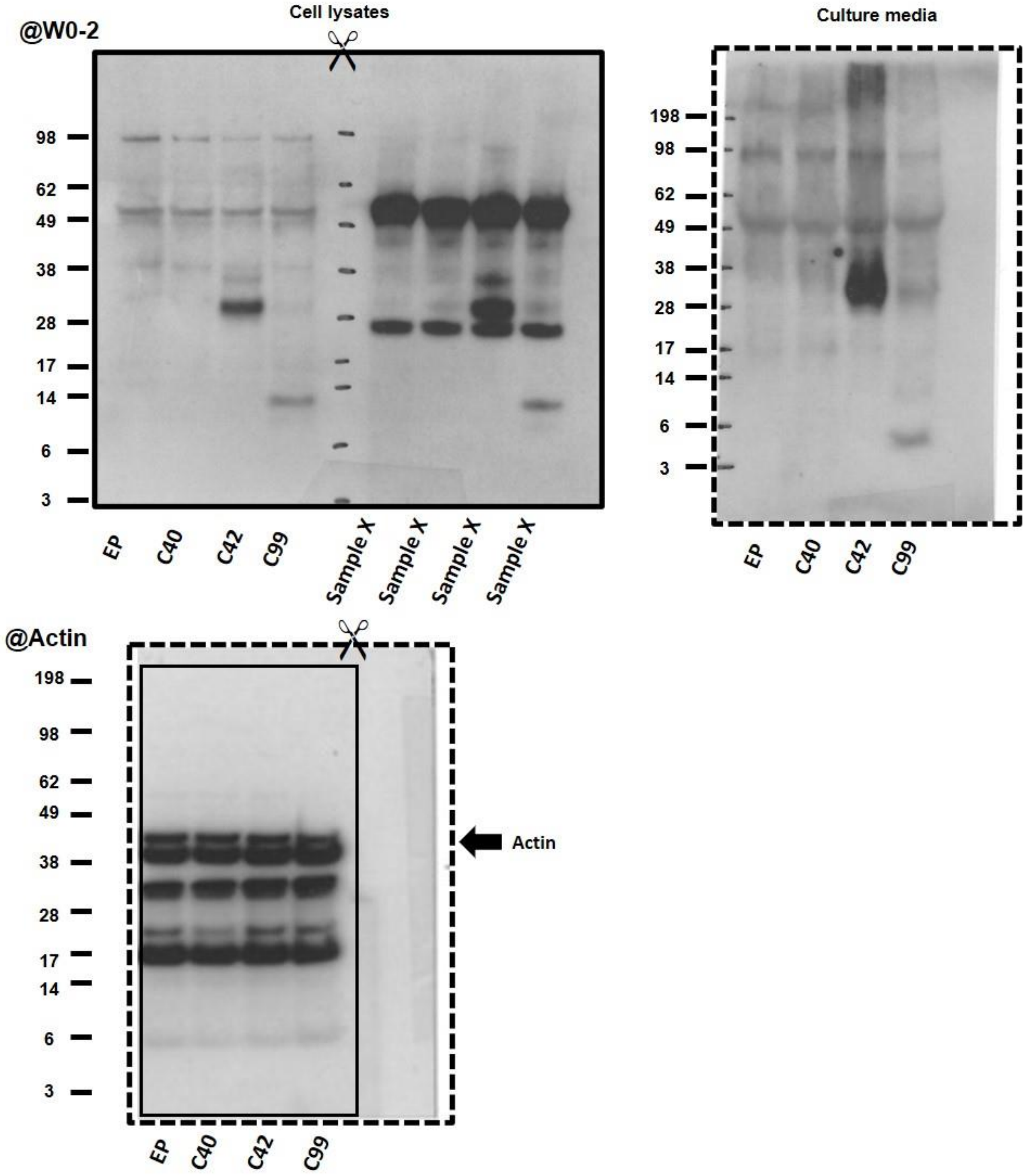

Supplemental Figure S9. Full length original gels of the cropped images presented in Figure 1b. Scissors indicate the membrane was cut before antibody binding and detection. A full-length Actin detection for samples presented in Figure 1b only are shown. A solid black line indicates the edges of the membrane. Dashed lines indicate that the detection films were trimmed prior to scanning, the edges of which have been included in the images where appropriate. Sample X are data not presented in the manuscript.

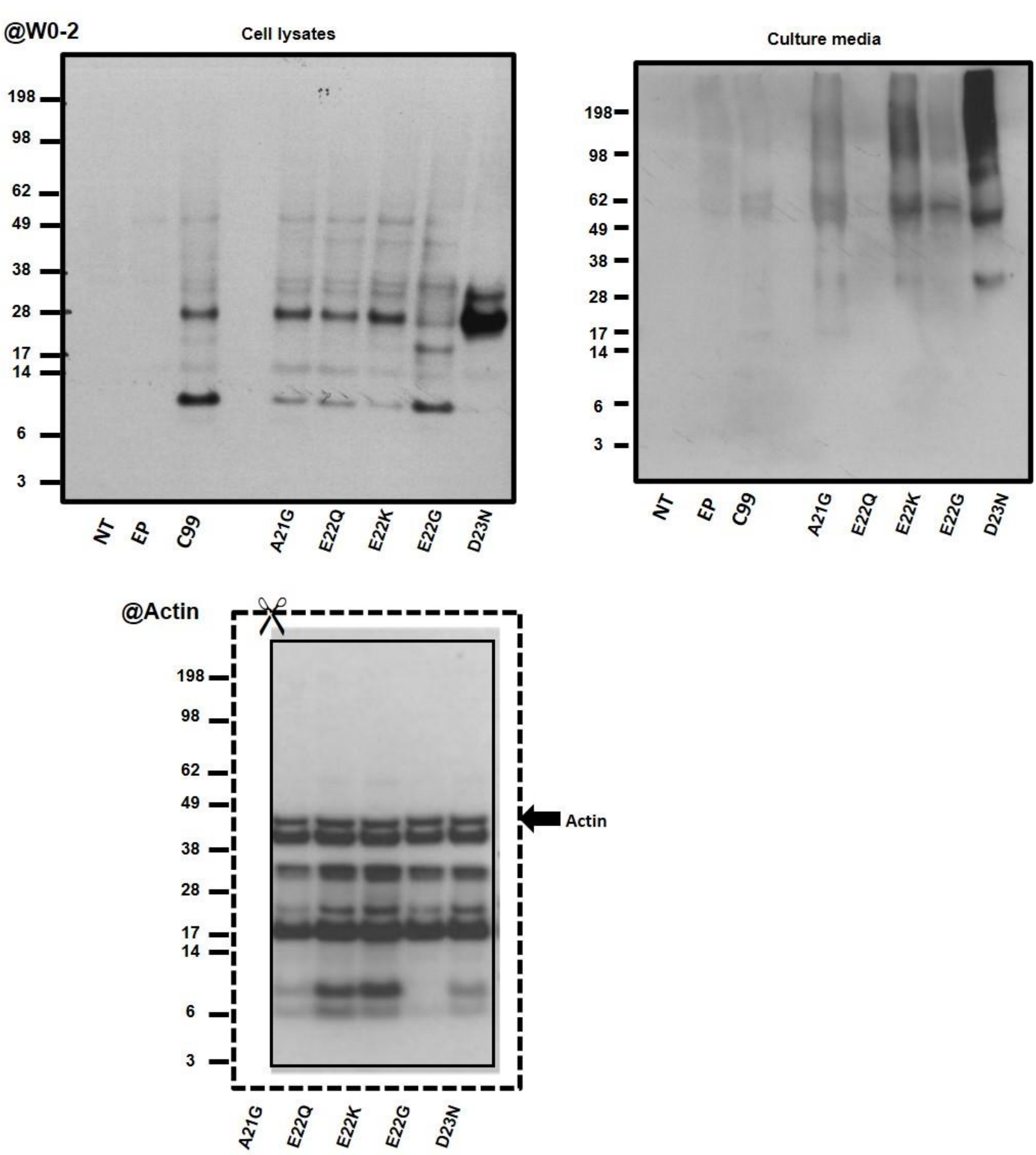

Supplemental Figure S10. Full length gels of the cropped images presented in Figure 1c. The scissors indicate the membrane was cut before antibody binding and detection. A full-length Actin detection for samples presented in Figure 1c only are shown and membrane edges are indicated by the solid black line. Dashed lines indicate that the detection films were trimmed prior to scanning, the edges of which have been included in the images where appropriate.

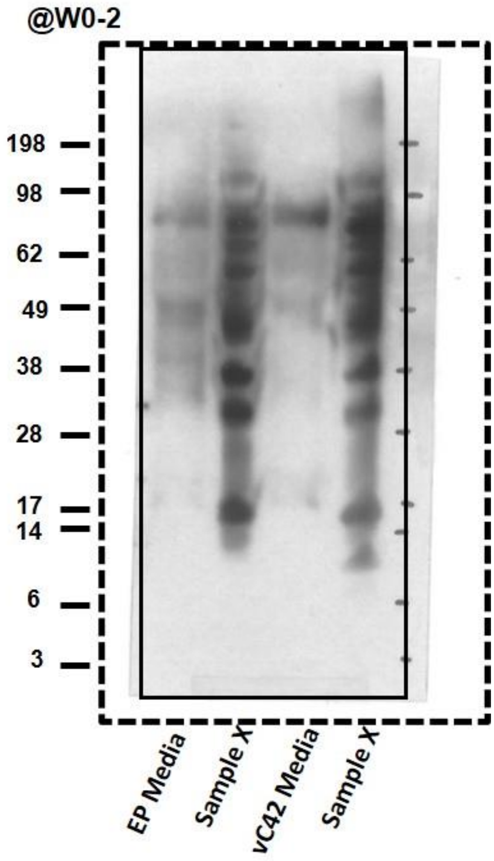

Supplemental Figure S11. Full length original gel of the cropped image presented in Figure 3, right panel. Sample X are data not presented in the manuscript. Dashed lines indicate the membrane was cut before antibody binding and detection. We provide the full-length image of gels for samples presented in Figure 3 only. The scissors indicate the membrane was cut before antibody binding and detection. A solid black line indicates the edges of the membrane. Dashed lines indicate that the detection film was trimmed prior to scanning, the edges of which have been included in the images where appropriate.

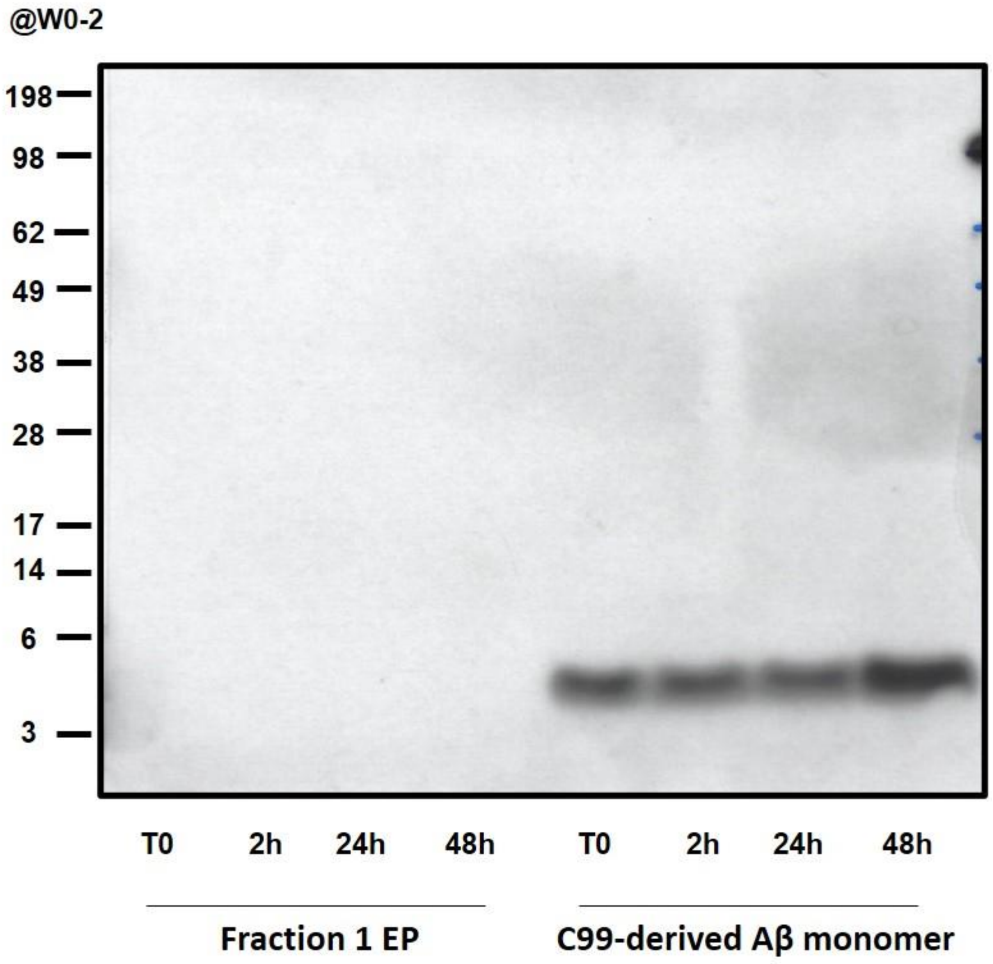

Supplemental Figure S12. Full length gel of the cropped image presented in Figure 4, top left panel.

	**Sequence**
C42	DAEFRHDSGYEVHHQKLVFFAEDVGSNKGAIIGLMVGGVVIA
vC42	DAEFRHDSGYEVHHQKLV**S**FAEDVGSNKGAIIGLMV**D**GVVIA

Supplemental Table S1. Primary sequence of the wild type (C42) and variant C42 (vC42) sequences

	Forward	Reverse
A21G	5'-ggtgttctttggagaagatgtgggttcaaac-3'	5'-cacatcttctccaaagaacaccaatttttgatg-3'
E22Q	5'-ggtgttctttgcacaagatgtgggttcaaac-3'	5'-cccacatcttgtgcaaagaacaccaatttttg-3'
E22K	5'-ggtgttctttgcaaaagatgtgggttcaaac-3'	5'-cccacatcttttgcaaagaacaccaatttttg-3'
E22G	5'-gtgttctttgcaggagatgtgggttcaaac-3'	5'-cccacatctcctgcaaagaacaccaatttttg-3'
D23N	5'-ggtgttctttgcagaaaatgtgggttcaaac-3'	5'-cccacattttctgcaaagaacaccaatttttg-3'

Supplemental Table S2. Primer sequences for site directed mutagenesis of C99 to introduce FAD mutations

	Forward	Reverse
GAPDH	5’-acccagaagactgtggatgg-3’	5’acacattgggggtaggaaca-3’
C40	5’ gctggaggatgcagaattccgac 3’	5’ cgcccaccatgagtccaatgattg 3’

Supplemental Table S3. Primer sequences for qPCR to probe for C40 transfection efficiency in CHO cells.
